# All‐Metal‐Organic Framework‐Derived Battery Materials on Carbon Nanotube Fibers for Wearable Energy‐Storage Device

**DOI:** 10.1002/advs.201801462

**Published:** 2018-10-11

**Authors:** Qichong Zhang, Zhenyu Zhou, Zhenghui Pan, Juan Sun, Bing He, Qiulong Li, Ting Zhang, Jingxin Zhao, Lei Tang, Zengxing Zhang, Lei Wei, Yagang Yao

**Affiliations:** ^1^ National Laboratory of Solid State Microstructures College of Engineering and Applied Sciences and Collaborative Innovation Center of Advanced Microstructures Nanjing University Nanjing 210093 China; ^2^ Division of Advanced Nanomaterials Key Laboratory of Nanodevices and Applications Joint Key Laboratory of Functional Nanomaterials and Devices CAS Center for Excellence in Nanoscience Suzhou Institute of Nano‐Tech and Nano‐Bionics Chinese Academy of Sciences Suzhou 215123 China; ^3^ Division of Nanomaterials Suzhou Institute of Nano‐Tech and Nano‐Bionics Nanchang Chinese Academy of Sciences Nanchang 330200 China; ^4^ School of Electrical and Electronic Engineering Nanyang Technological University 50 Nanyang Avenue Singapore 639798 Singapore; ^5^ Nano Science and Technology Institute University of Science and Technology of China Suzhou 215123 China; ^6^ Shanghai Key Laboratory of Special Artificial Microstructure Materials and Technology School of Physics Science and Engineering Tongji University Shanghai 200092 China

**Keywords:** aqueous rechargearable batteries, binder‐free electrodes, fibers, MOF‐derived battery materials, wearable electronics

## Abstract

The ever‐increasing demands for portable and wearable electronics continue to drive the development of high‐performance fiber‐shaped energy‐storage devices. Metal‐organic frameworks (MOFs) with well‐tunable structures and large surface areas hold great potential as precursors and templates to form porous battery materials. However, to date, there are no available reports about fabrication of wearable energy‐storage devices on the utilization of all‐MOF‐derived battery materials directly grown on current collectors. Here, MOF‐derived NiZnCoP nanosheet arrays and spindle‐like α‐Fe_2_O_3_ on carbon nanotube fibers are successfully fabricated with impressive electrochemical performance. Furthermore, the resulting all‐solid‐state fiber‐shape aqueous rechargeable batteries take advantage of large specific surface area and abundant reaction sites of well‐designed MOF‐derived electrode materials to yield a remarkable capacity of 0.092 mAh cm^−2^ and admirable energy density of 30.61 mWh cm^−3^, as well as superior mechanical flexibility. Thus, this research may open up exciting opportunities for the development of new‐generation wearable aqueous rechargeable batteries.

The rapid development of portable and wearable electronics has stimulated ever‐increasing demand for efficient energy‐storage technologies.^1^, ^2^, ^3^, ^4^, ^5^, ^6^, ^7^, ^8^, ^9^, ^10^ As an emerging class of energy supply devices for wearable electronics, fiber‐shaped energy‐storage devices have attracted significant scientific and technical interest due to their inherent advantages of light weight, compactness and easy weavability.^11^, ^12^, ^13^, ^14^, ^15^, ^16^, ^17^ Although fiber‐shaped supercapacitors feature the advantages of high power density, rapid charge/discharge capability and superior cyclability, their unstable discharge platform and low energy density are still serious hindrances for powering wearable energy‐consuming devices.^18^, ^19^, ^20^, ^21^, ^22^, ^23^, ^24^, ^25^ The construction of aqueous rechargeable batteries (ARBs) has proven an extremely effective strategy to achieve stable output voltage and high energy density.^26^, ^27^, ^28^, ^29^, ^30^, ^31^, ^32^, ^33^, ^34^, ^35^, ^36^ Noted that the performance of eletrode materials is very important for wearable energy storage devices.^37^, ^38^, ^39^, ^40^ Therefore, it is of crucial significance to synthesize highly capacitive electrode materials for fiber‐shaped ARBs (FARBs).

Metal‐organic frameworks (MOFs) have been identified as promising precursors and templates to form porous materials with well‐tunable structures and high surface areas.^41^, ^42^ Notably, these novel porous materials with large specific surface area and rich reaction sites are widely known as excellent electrochemically active materials.^43^, ^44^, ^45^ However, the above‐mentioned MOF‐derived porous structures are generally produced in powder form, which requires the use of the polymer binder and conductive addictive in the electrochemical reaction, leading to fewer active sites, loss of active materials, enlarged interfacial resistance, and poor flexibility. A feasible approach to address this issue is to directly grow MOF‐derived active materials on conductive substrates with better electrical and mechanical contact as binder‐free electrodes.^46^, ^47^, ^48^, ^49^, ^50^ To date, there has been no report of the fabrication of wearable ARBs based on the utilization of all‐MOF‐derived battery materials grown on current collectors.

Transition metal phosphides are ideal candidates for high‐performance battery materials due to their superior conductivity, impressive redox activity, low cost, and eco‐friendliness.^51^, ^52^, ^53^, ^54^, ^55^ Recent research demonstrates that metal‐ion doping can improve the electronic conductivity and electrochemical performance of battery materials.^20^, ^56^ Inspired by MOFs with tunable chemical compositions, we have designed and fabricated MOF‐derived three dimensionally well‐aligned NiZnCoP nanosheet arrays on carbon nanotube fiber (CNTF) as novel binder‐free cathode materials with enhanced electrochemical performance.

Among various anode materials, iron oxide (Fe_2_O_3_) has been intensively studied due to its high theoretical capacity, wide operating potential window, cost‐effectiveness natural abundance, and environment benignancy.^57^, ^58^, ^59^, ^60^, ^61^, ^62^, ^63^, ^64^ However, the low specific capacity and inferior rate performance, which result from the material's low conductivity and insufficient ionic diffusion rate, severely hinder its further application in FARBs. Developing nanostructure Fe_2_O_3_ with a larger active surface on a highly conductive fiber substrate is a plausible strategy to overcome this. Herein, we demonstrate a facile approach to directly grow MOF‐derived spindle‐like α‐Fe_2_O_3_ on oxidized carbon nanotube fiber (S‐α‐Fe_2_O_3_/OCNTF) with impressive electrochemical performance.

As a proof‐of‐concept demonstration, we successfully assembled wearable FARBs with a stable output voltage of 1.05 V by employing both MOF‐derived NiZnCoP@CNTF and S‐α‐Fe_2_O_3_/OCNTF as the cathode and anode. Benefiting from the large specific surface area and abundant reaction sites of the MOF‐derived battery materials, the resulting FARBs deliver a remarkable specific capacity of 0.092 mAh cm^−2^ and impressive energy density of 30.61 mWh cm^−3^.

The fabrication process of our twisted FARB device is schematically depicted in **Figure** **1** and the details are presented in the Experimental section. First, MOF‐derived NiZnCoP nanosheet arrays were directly grown on the CNTF (NiZnCoP/CNTF) as a novel cathode through a three‐step process consisting of MOF growth, subsequent anion exchange/etching, and finally phosphorization treatment in argon. Simultaneously, the MOF‐derived S‐α‐Fe_2_O_3_/OCNF as an anode was fabricated via a solvothermal method and postannealing in air. Thereafter, both the as‐fabricated NiZnCoP/CNTF and S‐α‐Fe_2_O_3_/CNTF electrodes were coated with a thin layer of KOH/polyvinyl alcohol (PVA) gel electrolyte. Finally, the FARB device was successfully assembled by twisting the two electrodes together and leaving it overnight until the electrolyte was fully solidified.

**Figure 1:**
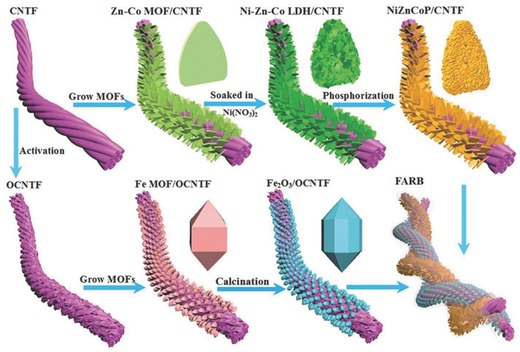
Schematic fabrication process of the twisted FARB device.

The scanning electron microscopy (SEM) image in **Figure** **2**a reveals that the Zn‐Co MOF nanosheet arrays were uniformly grown on the entire surface of CNTF via a modified solution synthesis strategy. The enlarged SEM image in Figure 2b clearly illustrates that the Zn‐Co MOF nanosheet arrays are densely packed and highly ordered on the hybrid fiber. The Co MOF, which has a similar morphology, is shown in Figure S1 (Supporting Information). After reacting with Ni(NO_3_)_2_ ethanol solution, small nanoflakes were homogeneously anchored onto the surfaces of the as‐fabricated Zn‐Co MOF and Co MOF nanosheet arrays, as presented in Figure 1c and Figure S2 in the Supporting Information, thus forming hierarchical Co MOF@Co‐Ni layered double hydroxide (LDH) and Zn‐Co MOF@Ni‐Zn‐Co LDH core–shell heterostructures. The as‐obtained Co MOF@Co‐Ni LDH and Zn‐Co MOF@Ni‐Zn‐Co LDH were further annealed with NaH_2_PO_2_ at 350 °C for 2 h under argon atmosphere, and the corresponding SEM images are shown in Figure 2d, e. Interestingly, the Zn‐Co MOF@Ni‐Zn‐Co LDH‐derived NiZnCoP nanosheet arrays, which were synthesized through preintroducing elemental Zn into the MOF structure, retained the nanosheet morphology, in contrast with the Co MOF@Ni‐Co LDH‐derived NiCoP nanosheet arrays. Furthermore, the surfaces of the NiZnCoP nanosheets became rough (Figure 2e, f) due to the formation of massive nanoparticles. A magnified transmission electron microscopy (TEM) image depicted in Figure 2f reveals that NiZnCoP nanosheet is loaded with abundant nanocrystalline, which is in good agreement to SEM image (Figure 2e), leading to a large number of reaction sites derived from small‐sized effects on its surfaces. Simultaneously, the high‐magnification TEM image shows a relatively uniform distribution of small‐sized nanoparticles with a size range of 3.5–7.5 nm. The mesoporous nanoarchitecture facilitates the transport and diffusion of electrolyte, resulting in the rapid charge transfer reactions. This architecture can be presumed to have increased the surface areas of the active materials and facilitated fast ion and electron transportation, which is beneficial to the electrochemical performance. To confirm this, the Brunauer–Emmett–Teller (BET) surface areas of the NiZnCoP is estimated to be 81.7 m^2^ g^−1^ (Figure S3 in the Supporting Information). Additionally, the X‐ray diffraction (XRD) patterns of Zn‐Co MOF, Zn‐Co MOF@Ni‐Zn‐Co LDH, and NiZnCoP are illustrated in Figure S4 (Supporting Information). The chemical compositions and valence states of the NiZnCoP were investigated with X‐ray photoelectron spectroscopy (XPS) (Figure S5 in the Supporting Information). The energy‐dispersive spectroscopy (EDS) mapping results of the NiZnCoP in Figure 2g clearly suggest the homogeneous distribution of the elements Zn, Ni, Co, P, N, and C across the entire nanosheet; note that the N and C come from the carbonization of organic ligands in the MOF precursor. To further analyze the elemental composition of the as‐fabricated NiZnCoP nanosheet, the EDS spectrum was recorded. The results, depicted in Figure 2h, again clearly prove the coexistence of the elements Zn, Ni, Co, and P.

**Figure 2:**
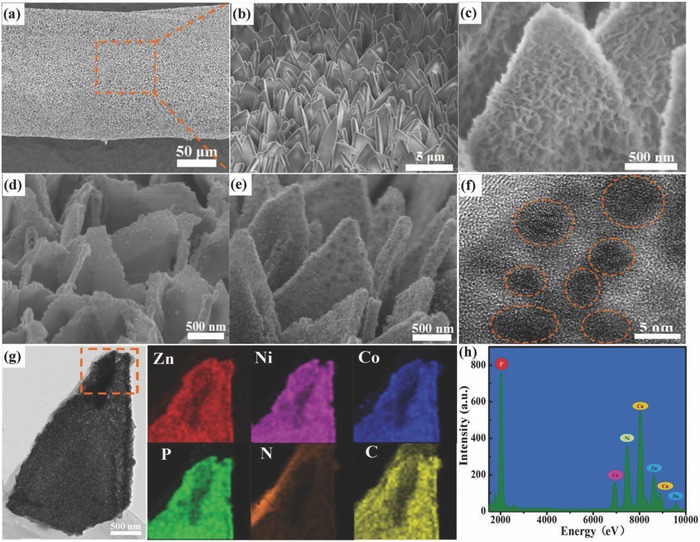
Characterization of cathodes. a,b) SEM images of Zn‐Co MOF nanosheet arrays on CNTF at increasing magnifications. c) SEM image of Ni‐Zn‐Co LDH nanosheet arrays. d) SEM image of NiCoP nanosheet arrays. e) SEM image of NiZnCoP nanosheet arrays. f) High‐magnification TEM image of the NiZnCoP nanosheet. g) Low‐magnification TEM image and the corresponding EDS element mappings of Zn, Ni,Co,P, N, and C in the NiZnCoP nanosheet. h) EDS spectrum of NiZnCoP nanosheet.

Benefiting from the above‐described structural and compositional features, an enhanced electrochemical performance was expected for the NiZnCoP/CNTF electrodes. The electrochemical performance of the as‐fabricated fibrous electrode materials was first investigated by using the fibrous samples as working electrodes in a three‐electrode system. **Figure** **3**a compares the cyclic voltammetry (CV) curves for the pristine CNTF, NiCoP/CNTF, and NiZnCoP/CNTF electrodes measured at a scan rate of 10 mV s^−1^. Evidently, the pristine CNTF shows a much smaller current than the NiZnCoP/CNTF and NiCoP/CNTF, indicating that the CNTF itself makes a negligible contribution to the capacity of the hybrid fibrous electrode. As expected, the as‐fabricated NiZnCoP/CNTF electrode exhibits a larger CV curve area and stronger redox peak than NiCoP/CNTF, implying a dramatically improved specific capacity and faster redox reaction. As shown in Figure 3b, the NiZnCoP/CNTF electrode presents a much longer discharging time than the NiCoP/CNTF electrode at the same current density, presumably arising from the unique structure and composition, further demonstrating its enhanced electrochemical performance. The function of Zn, Ni, and P element is further revealed in Figure S6 (Supporting Information). Figure 3c illustrates the CV curves of the NiZnCoP/CNTF electrode at different scan rates from 2 to 10 mV s^−1^. Notably, a pair of redox peaks is clearly observed, demonstrating the presence of redox reactions of NiZnCoP during the electrochemical process. The discharge curves of the NiZnCoP/CNTF electrode at different current densities, shown in Figure 3d, clearly demonstrate that the well‐defined discharge plateaus is around 0.3 V with small shift to lower potential at high discharge current density. The specific capacities were calculated from the discharge curves and the corresponding results are presented in Figure 3e. Specifically, the specific capacity of the NiZnCoP/CNTF electrode is as high as 0.24 mAh cm^−2^ at a current density of 1 mA cm^−2^, which is about two times that of the NiCoP/CNTF electrode, and 86.8% of the initial specific capacity is well retained at a high discharge current density of 10 mA cm^−2^, demonstrating its excellent rate performance. As shown in Figure 3f and Table S1 in the Supporting Information, the *R*_S_ and *R*_CT_ have been largely reduced by introducing the Zn during the phosphating process. In addition, it can be clearly observed that the slope of the NiZnCoP/CNTF is larger than that of NiCoP/CNTF in low frequency region, illustrating efficient ions diffusion of the electrolyte in NiZnCoP/CNTF during redox reaction. Figure S7 in the Supporting Information demonstrates that NiZnCoP/CNTF retained 92.1% of its initial capacity after long‐term charge/discharge cycling at 3 mA cm^−2^ for 8000 cycles. These superior properties of the hierarchical NiZnCoP/CNTF electrode make it a very attractive cathode material for high‐performance wearable ARBs.

**Figure 3:**
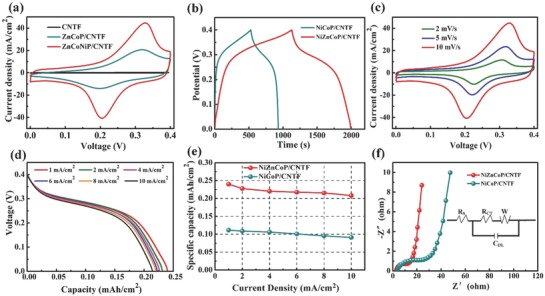
Electrochemical performance of cathodes. a) Comparison of CV curves of pristine CNTF, NiCoP/CNTF, and NiZnCoP/CNTF electrodes measured at a scan rate of 10 mV s^−1^. b) Comparison of GCD curves of NiCoP/CNTF and NiZnCoP/CNTF obtained at a current density of 1 mA cm^−2^. c) CV curves of the NiZnCoP/CNTF electrode at different scan rates. d) Discharge curves of the NiZnCoP/CNTF electrode at different current densities. e) The specific capacities of the NiCoP/CNTF and NiZnCoP/CNTF electrodes at different current densities. f) The EIS curves of the NiCoP/CNTF and NiZnCoP/CNTF electrodes.

To achieve a high‐performance anode material, an MOF, MIL‐88‐Fe, was uniformly grown on the OCNTF surface by solvothermal synthesis, as shown in **Figure** **4**a. The XRD pattern of as‐fabricated MIL‐88‐Fe is illustrated in Figure S8 (Supporting Information). An SEM image of the OCNTF before MOF growth is presented in Figure S9 (Supporting Information). The high‐magnification SEM image in Figure 4b illustrates that the MIL‐88‐Fe grew in the form of regular dodecahedron‐shaped single crystals. After the subsequent annealing treatment, the MIL‐88‐Fe sample was completely transformed to spindle‐like α‐Fe_2_O_3_ (Figure 4c). The microstructure of S‐α‐Fe_2_O_3_ was further analyzed using TEM. The low‐magnification TEM image and corresponding EDS mapping results (Figure 4d) clearly reveal the homogeneous distribution of Fe, O, and C across the entire S‐α‐Fe_2_O_3_ sample, where the C signal is attributed to the organic compounds from the MIL‐88‐Fe precursor being annealed. The EDS spectrum, shown in Figure 4e, clearly demonstrates the coexistence of Fe and O in the as‐fabricated S‐α‐Fe_2_O_3._ Furthermore, the inset of Figure 4e shows that the atomic ratio of Fe:O is close to 2:3, again confirming that the spindle‐like material is composed of Fe_2_O_3_. The representative high‐resolution TEM image in Figure 4f demonstrates that the lattice fringes have a spacing of 0.28 and 0.21 nm, which agrees well with the (104) and (113) plane of α‐Fe_2_O_3_. In the XRD pattern, presented in Figure 4g, all of the diffraction peaks can be well indexed to α‐Fe_2_O_3_ (JCPDS card No. 33–0064). The Raman spectrum of the as‐fabricated S‐α‐Fe_2_O_3_ was recorded and is shown in Figure S10 (Supporting Information). The chemical compositions and valence states of the S‐α‐Fe_2_O_3_ were further investigated with XPS and the corresponding results are shown in Figure 4h, i. The Fe 2p XPS spectrum shows two distinct peaks at 711.9 and 725.4 eV, corresponding to the Fe 2p_3/2_ and Fe 2p_1/2_ spin‐orbit peaks, respectively, whereas the peaks in the O 1s band at 530.7 and 532.8 eV correspond to Fe–O and C–O, respectively, which agree well with the previous reports of α‐Fe_2_O_3._ The BET test results demonstrate that and the surface area of the α‐Fe_2_O_3_ is estimated to be 106.5 m^2^ g^−1^ (Figure S11 in the Supporting Information). All of the above‐mentioned data verify the successful fabrication of S‐α‐Fe_2_O_3_/CNTF, which is expected to be applied in high‐performance FARBs.

**Figure 4:**
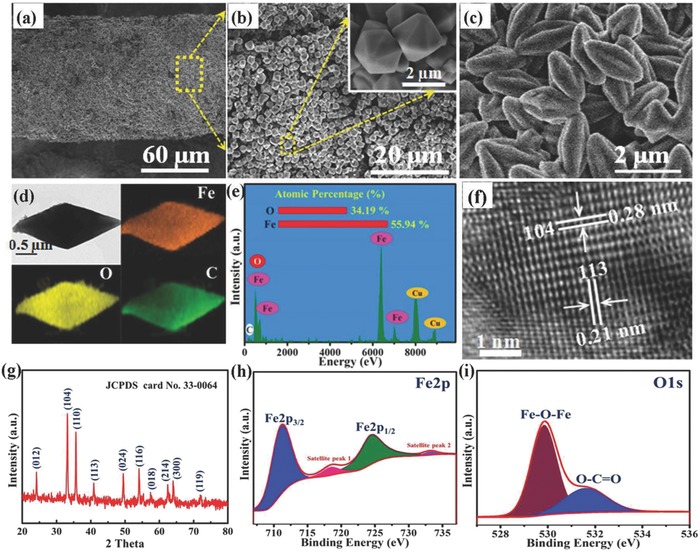
Characterization of anodes. a,b) SEM images of MIL‐88‐Fe/OCNTF at increasing magnifications. c) SEM image of S‐α‐Fe_2_O_3_/OCNTF. d) Low‐magnification TEM image and the corresponding EDS element mappings for Fe, O, and C in the S‐α‐Fe_2_O_3_. e) EDS spectrum of S‐α‐Fe_2_O_3_. f) High‐magnification TEM image of the S‐α‐Fe_2_O_3_. g) XRD spectrum of the S‐α‐Fe_2_O_3_. XPS survey scan of h) Fe2p and i) O1s.

To evaluate the electrochemical properties of the as‐synthesized anode materials, the S‐α‐Fe_2_O_3_/OCNTF was measured in a three‐electrode system using 3 m KOH aqueous solution as the electrolyte. **Figure** **5**a compares the CV curves of the pristine CNTF, OCNTF, S‐α‐Fe_2_O_3_/CNTF, and S‐α‐Fe_2_O_3_/OCNTF electrodes collected at 10 mV s^−1^. Clearly, the S‐α‐Fe_2_O_3_/OCNTF electrode curve has a larger area than the other electrodes, indicating high specific capacity. Importantly, the S‐α‐Fe_2_O_3_/OCNTF electrode exhibits a much higher specific capacity than the S‐α‐Fe_2_O_3_/CNTF electrode, which is ascribed to the much greater mass loading of electrochemical active materials on OCNTF than CNTF. The CV curves of the S‐α‐Fe_2_O_3_/OCNTF electrode with scan rates ranging from 5 to 30 mV s^−1^ in the potential window of −1.3 to 0 V (Figure 5b) show a pair of obvious redox peaks, implying typical battery properties. This is further supported by the obvious discharge plateau in Figure 5c. As presented in Figure 5d, the specific capacity of the S‐α‐Fe_2_O_3_/OCNTF electrode is as high as 0.21 mAh cm^−2^ at a current density of 1 mA cm^−2^. Even when the current density was increased to 10 mA cm^−2^, this value still reached 0.13 mAh cm^−2^, indicating an exceptional rate capability. In addition, as shown in Figure S12 (Supporting Information), 90.2% specific capacity is retained after long‐term charging/discharging cycling at 3 mA cm^−2^ for 4000 cycles. These results suggest that S‐α‐Fe_2_O_3_/OCNTF is a promising anode material for applications in next‐generation wearable energy‐storage devices.

**Figure 5:**
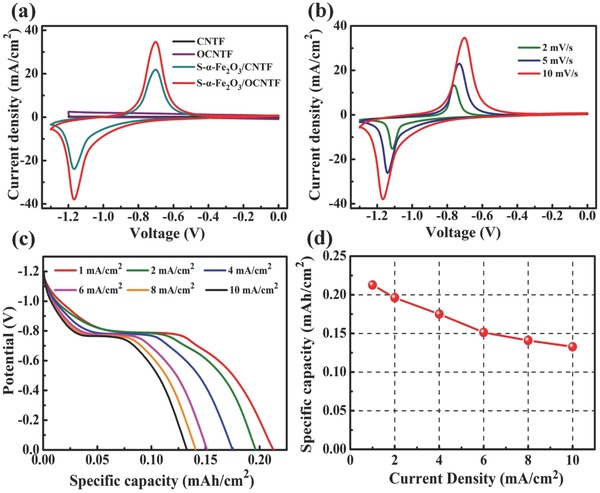
Electrochemical performance of anodes. a) Comparison of CV curves of pristine CNTF, OCNTF, S‐α‐Fe_2_O_3_/CTNF, and S‐α‐Fe_2_O_3_/OCTNF electrodes measured at a scan rate of 10 mV s^−1^. b) CV curves of S‐α‐Fe_2_O_3_/OCTNF at different scan rates. c) Discharge curves of the S‐α‐Fe_2_O_3_/OCTNF electrode at different current densities. d) The specific capacities of the S‐α‐Fe_2_O_3_/OCTNF electrode at different current densities.

**Figure** **6**a comparatively demonstrates the CV curves of the as‐obtained S‐α‐Fe_2_O_3_/OCNTF and NiZnCoP@CNTF electrodes at 10 mV s^−1^ and it should be noted that both the electrodes consist of a couple of prominent redox peaks. It is thus anticipated to develop a high‐voltage aqueous full cell. Consequently, a prototype twisted FARB was successfully assembled using KOH‐PVA as a solid electrolyte and two obvious redox peaks are presented in Figure 6b. The low‐magnification SEM of the as‐assembled device is shown in Figure S13 in the Supporting Information. The GCD curves of the FARB device in Figure 6c exhibit characteristic charge and discharge plateaus at ≈1.35 and ≈1.05 V, respectively. More importantly, a high specific capacity of 0.092 mAh cm^−2^ was achieved for our FARB at a current density of 1 mA cm^−2^. The energy and power densities are important parameters with respect to energy‐storage device performance. Here, a peak energy density of 30.61 mWh cm^−3^ was obtained together with a maximum power density of 3339.7 mW cm^−3^ (Figure 6e). These values considerably exceed previously reported fiber‐shaped energy‐storage devices, including Ni//Zn batteries,^15^ NiCo//Zn batteries,^16^ Co//Zn batteries,^17^ Co_3_O_4_/Ni wire//reduced graphene oxide/carbon fiber asymmetric supercapacitors,^24^ and MoS_2_‐reduced graphene oxide/CNTF//reduced graphene oxide/CNTF asymmetric supercapacitors.^25^ The long‐term cycling stability of our FARB was further studied at 2 mA cm^−2^ (Figure 6e). Our device exhibits excellent stability with 88.6% retention of the initial capacity after 3000 cycles. The Nyquist plot of the electrochemical impedance of our FARB device is illustrated in Figure S14 (Supporting Information). To demonstrate the potential application of the assembled FARB in portable and wearable electronics, we also performed a series of mechanical flexibility tests. The GCD curves of the assembled FARB device, shown in Figure 6f, nearly overlap completely at different bending angles ranging from 0° to 180°, indicating that the device possesses excellent mechanical stability. Furthermore, a LED can be illumined by the two series‐connected FARBs device at different bending angles, and the brightness of LEDs is almost constant (Figure S15 in the Supporting Information), again confirming the outstanding flexibility of our FARBs. As shown in Figure S16 (Supporting Information), the brightness of the LED is well retained when the FARBs device is bended at 90° for for 100 cycles. More importantly,our FARBs device were woven into flexible power textile presented in Figure S17 (Supporting Information) to demonstrate their remarkable weavability.

**Figure 6:**
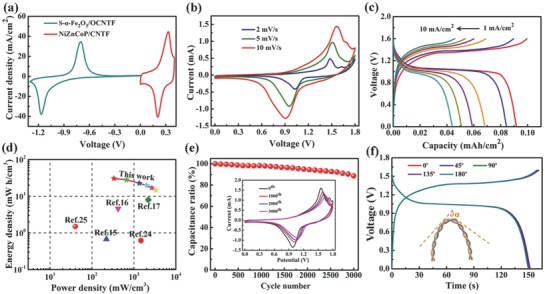
Electrochemical performance and flexibility test of the assembled FARBs. a) Comparison CV curves of S‐α‐Fe_2_O_3_/OCNTF anode and NiZnCoP@CNTF cathode at a scan rate of 10 mV s^−1^. b) CV curves of the assembled FARBs measured at different scan rates. c) GCD curves collected at different current densities. d) Volumetric energy and power densities of the assembled FARBs compared with previously reported fiber‐shaped energy‐storage devices. e) Cycling performance of the as‐assembled FARBs. (The inset are CV curves at different cycles.) f) GCD curves of the as‐assembled FARBs bent at various angles at a current density of 2 mA cm^−2^.

In summary, a prototype wearable FARB using all‐MOF‐derived battery materials on CNTFs as electrode materials was demonstrated for the first time. The well‐designed MOF‐derived NiZnCoP@CNTF and S‐α‐Fe_2_O_3_/OCNTF binder‐free electrodes integrated the features of large specific surface area, rich reaction sites, and short electron/ion diffusion path, thus resulting in prominent electrochemical performance. The electrochemical results demonstrate that our FARB delivers a remarkable capacity of 0.092 mAh cm^−2^ and an admirable energy density of 30.61 mWh cm^−3^. Additionally, our device show only negligible capacity decay after bending 3000 times, which indicates outstanding mechanical flexibility. This work may be a stepping stone toward the design and fabrication of next‐generation wearable energy‐storage devices.

## Experimental Section

*Synthesis MOF‐Derived NiZnCoP Nanosheet Arrays on CNTFs*: The Zn‐Co MOF on the CNTFs was fabricated using a modified solution method. Before the growth of MOFs, CNTFs were treated in oxygen plasma for 20 min at 150 W. Typically, 40 mL aqueous solution containing 2 mmol Co(NO_3_)_2_·6H_2_O and 1 mmol Zn(NO_3_)_2_·6H_2_O and 40 mL aqueous solution containing 20 mmol 2‐methylimidazole were mixed together under magnetic stirring and the pretreated CNTFs were immersed in the mixing solution. After 4 h of reaction at the room temperature, the Zn‐Co MOF/CNTF was washed with demonized water several times and dried at 60 °C in vacuum overnight. Then, the synthesized Zn‐Co MOF/CNTF was placed into a 100 mL ethanol solution containing 0.3 g Ni(NO_3_)_2_·6H_2_O for 30 min to obtain Zn‐Co MOF@Zn‐Co‐Ni LDH/CNT and followed by washing with ethanol and drying at 60 °C in vacuum overnight. Finally, the as‐obtained Zn‐Co MOF@Co‐Zn‐Ni LDH/CNTF was further annealed with NaH_2_PO_2_ at 350 °C for 2 h under argon atmosphere with the heating rate of 5 °C min^−1^. For comparison, Co MOF/CNTF was also fabricated using the same procedure as the Zn‐Co MOF/CNTF by replacing 1 mmol Zn(NO_3_)_2_·6H_2_O with 1 mmol Co(NO_3_)_2_·6H_2_O.

*Synthesis MOF‐Derived S‐α‐Fe_2_O_3_ on OCNTFs*: The OCNTF was prepared via a standard three‐electrode system, where 0.8 M H_2_SO_4_ aqueous solution as the electrolyte, Ag/AgCl electrode as the reference electrode, and a graphite sheet as the counter electrode. The oxidized process was performed with the CV method between 1 and 2 V, the scan rate was 25 mV s^−1^, and the cyclic number was 15. The as‐activated CNTFs were then rinsed several times with demonized water and air‐dried at 60 °C. Thereafter, 1.095 g ferric chloride and 0.748 g terephthalic acid were dissolved in 40 mL of DMF solution and stirred vigorously to form a homogeneous solution. The solution was then transferred into a 50 mL Teflon‐lined stainless steel autoclave and the OCNTFs were immersed in the solution. The autoclave was then sealed and maintained at 100 °C in an oven for 15 h. After cooling to the room temperature, the obtained OCNTFs were washed with distilled water and dried overnight at 60 °C. Finally, they were annealed into S‐α‐Fe_2_O_3_/OCNTF at 350 °C in air for 2 h with a heating rate of 2 °C min^−1^.

*Assembly of S‐α‐Fe_2_O_3_/OCNTF//NiZnCoP@CNTF FARBs*: The KOH‐PVA gel electrolyte was prepared by mixing 11.2 g KOH and 10 g PVA in 100 mL distilled water under vigorous stirring at 95 °C for 2 h until the solution became clear. The as‐fabricated FARBs were assembled using S‐α‐Fe_2_O_3_/OCNTF anodes, NiZnCoP@CNTF cathodes and KOH‐PVA gel electrolyte. S‐α‐Fe_2_O_3_/OCNTF and NiZnCoP@CNTF were then soaked in KOH‐PVA gel electrolyte for 30 min and annealed at 70 °C for 30 min. Finally, the FARB device was prepared successfully by twisting two electrodes together and leaving it overnight until the electrolyte was solidified.

## Conflict of Interest

The authors declare no conflict of interest.

## Supporting information

Supplementary
