# Supplementary material for: All‐Metal‐Organic Framework‐Derived Battery Materials on Carbon Nanotube Fibers for Wearable Energy‐Storage Device
Source: Adv Sci (Weinh). 2018 Oct 11;5(12):1801462. doi: 10.1002/advs.201801462 (PMC6299715; doi:10.1002/advs.201801462)
Supplement: Supplementary file 1 — Supplementary [file ADVS-5-1801462-s001.pdf]

Copyright WILEY-VCH Verlag GmbH & Co. KGaA, 69469 Weinheim, Germany, 2018.

ADVANCED SCIENCE
Open Access

Supporting Information

for *Adv. Sci.,* DOI: 10.1002/advs.201801462

All-Metal-Organic Framework-Derived Battery Materials on Carbon Nanotube Fibers for Wearable Energy-Storage Device

Qichong Zhang, Zhenyu Zhou, Zhenghui Pan, Juan Sun, Bing He, Qiulong Li, Ting Zhang, Jingxin Zhao, Lei Tang, Zengxing Zhang, Lei Wei, and Yagang Yao**

Copyright WILEY-VCH Verlag GmbH & Co. KGaA, 69469 Weinheim, Germany, 2018.
Supporting Information

Title: **All Metal-Organic Framework Derived Battery Materials on Carbon Nanotube Fibers for Wearable Energy Storage Device**

Qichong Zhang[+] Zhenyu Zhou[+], Zhenghui Pan, Juan Sun, Bing He, Qiulong Li, Ting Zhang, Jingxin Zhao, Lei Tang, Zengxing Zhang, Lei Wei and Yagang Yao**

Characterizations of materials.

The morphologies of the samples were characterized with a scanning electron microscope (Hitachi S-4800, 5 kV). X-ray diffraction patterns were obtained with a Rigaku D/MAX2500 V with Cu Kα radiation (λ = 1.5418 Å). X-ray photoelectron spectroscopy was recorded on an ESCALab MKII X-ray photoelectron spectrometer with non-monochromatized Mg Kα X-rays as the excitation source. High-resolution TEM images were recorded on an FEI Tecnai G2 20 high-resolution transmission electron microscope at an acceleration voltage of 200 kV. The samples were characterized by Raman spectroscopy (Raman, Labram HR 800) with a laser excitation of 532 nm. To measure the surface area of the electrode materials, nitrogen adsorption-desorption isotherms were measured on a Micromeritics ASAP 2020 at 77 K.

$$S= \pi \times d \times L \qquad (1)$$

$$S= \pi d^2 L/4 \qquad (2)$$

Where S, V represents the total area (cm^2) and volume (cm^3) of the device, d is the diameter of the device, *L* is the length of the decice.

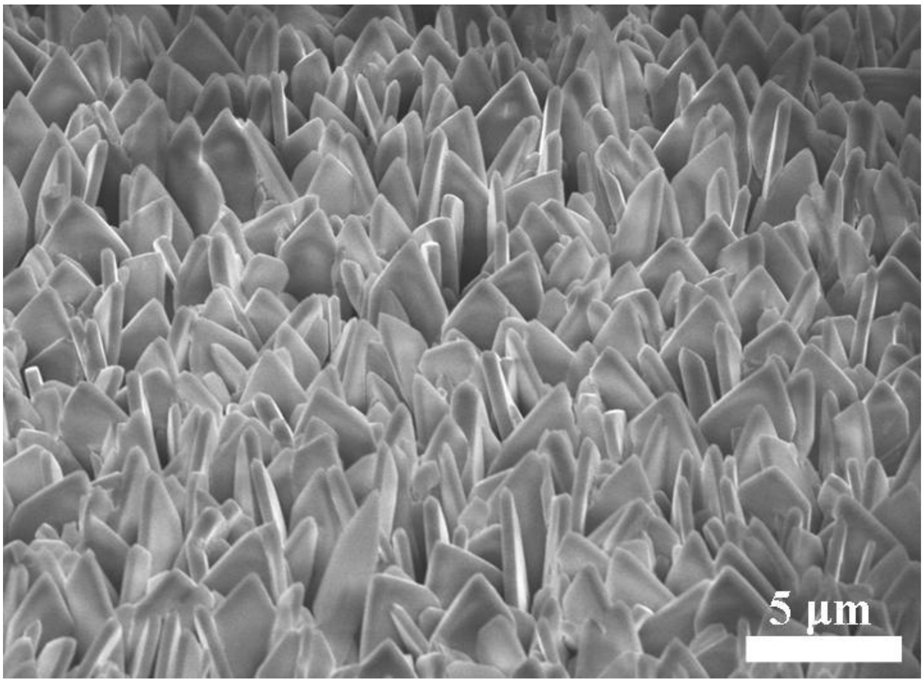

Figure S1. SEM image of Co MOF nanosheet arrays.

.

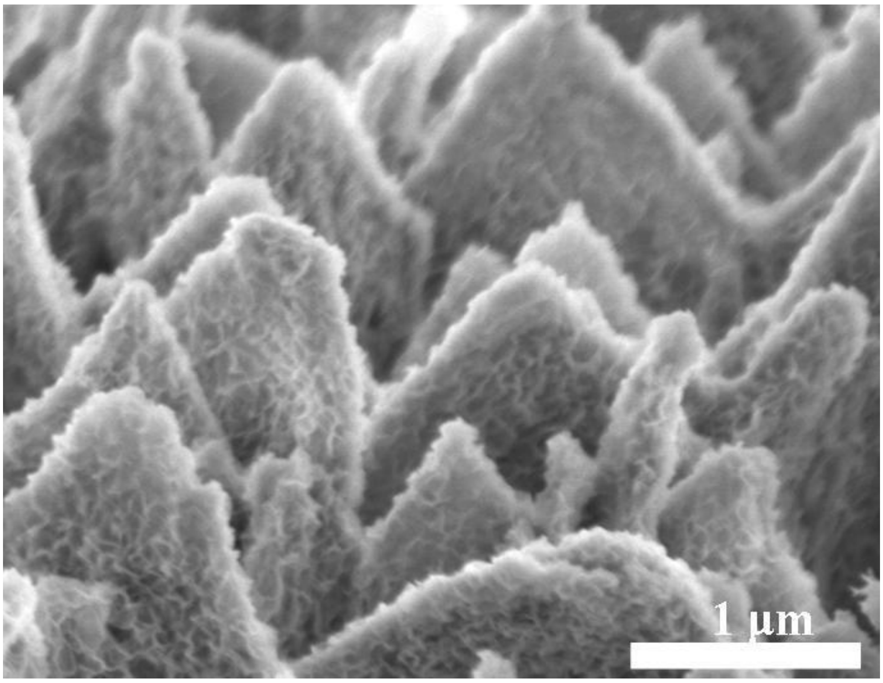

Figure S2. SEM image of Ni-Co LDH nanosheet arrays .

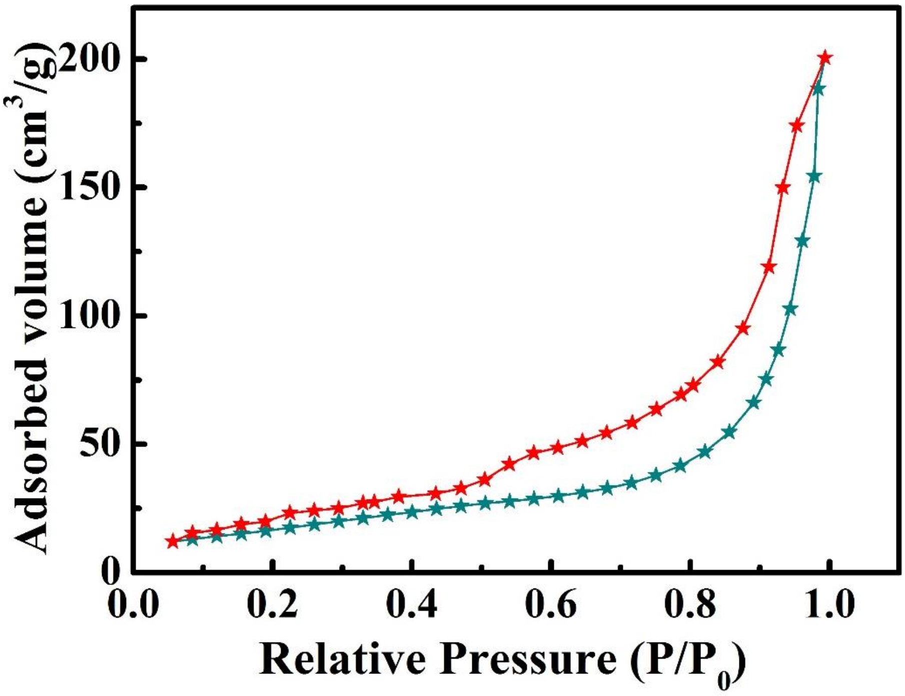

Figure S3. N_2 absorption-desorption isotherm of the NiZnCoP electrode materials.

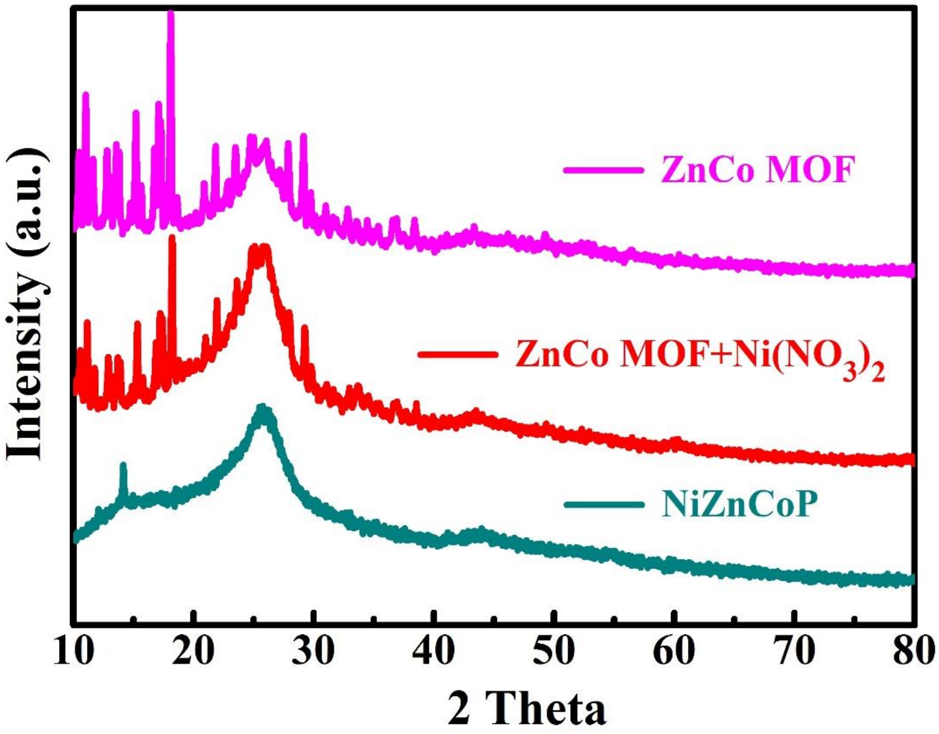

Figure S4. The XRD patterns of Zn-Co MOF, Zn-Co MOF@Ni-Zn-Co LDH and NiZnCoP.

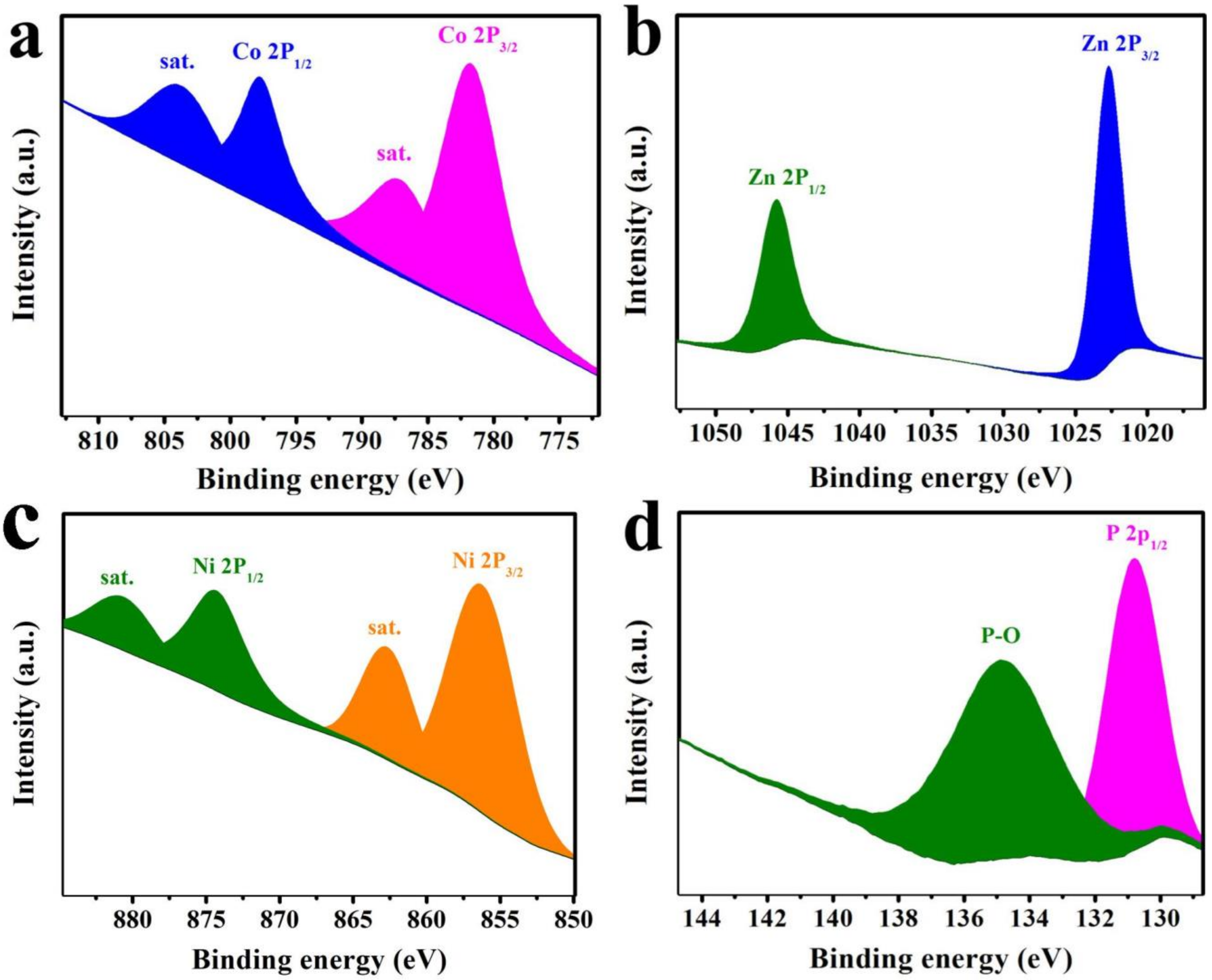

Figure S5. XPS spectra of the ZCNP NWAs. (a) Co 2p, two major peaks centred at 796.9 and 781.0 eV were identified as Co $2p_{1/2}$ and Co $2p_{3/}2$ spin-orbit peaks, respectively. (b) Zn 2p, the strong peaks located at 1022.7 and 1045.8 eV correspond to Zn $2p_{3/2}$ and Zn $2p_{1/2}$, respectively. (c) Ni 2p, two major peaks with binding energies at 874.2 and 856.2 eV, corresponding to the Ni $2p_{1/2}$ and Ni $2p_{3/2}$ spin-orbit peaks, respectively. (d) P 2p, 130.5 eV is assigned to P $2p_{1/2}$, and the peak at 134.6 eV is derived from P-O bonds.

Table S1 Fitting results for Electrochemical Impedance Spectroscopy

Samples	R_S (Ω)	R_{CT} (Ω)	C_{DL}	W
NiCoP/CNTF	3.57	36.3	0.78321	0.78542
NiZnCoP/CNTF	2.92	17.6	0.76223	0.68852

As shown in the inset of the revised Figure 3f, the impedance data were analyzed by fitting to an equivalent circuit consisting of the series resistance (R_S), double layer capacitance (C_{DL}), charge transfer resistance (R_{CT}) and Warburg behavior (W). At high frequency range, the value of the intercept at the real axis is used to estimate the R_S of the electrodes, which is the combination of the ionic resistance of the electrolyte and the resistance of the electrode material itself. The semicircle diameter in the plot corresponds to the R_{CT}, corresponding to the total resistance at the interface between the electrode and the electrol

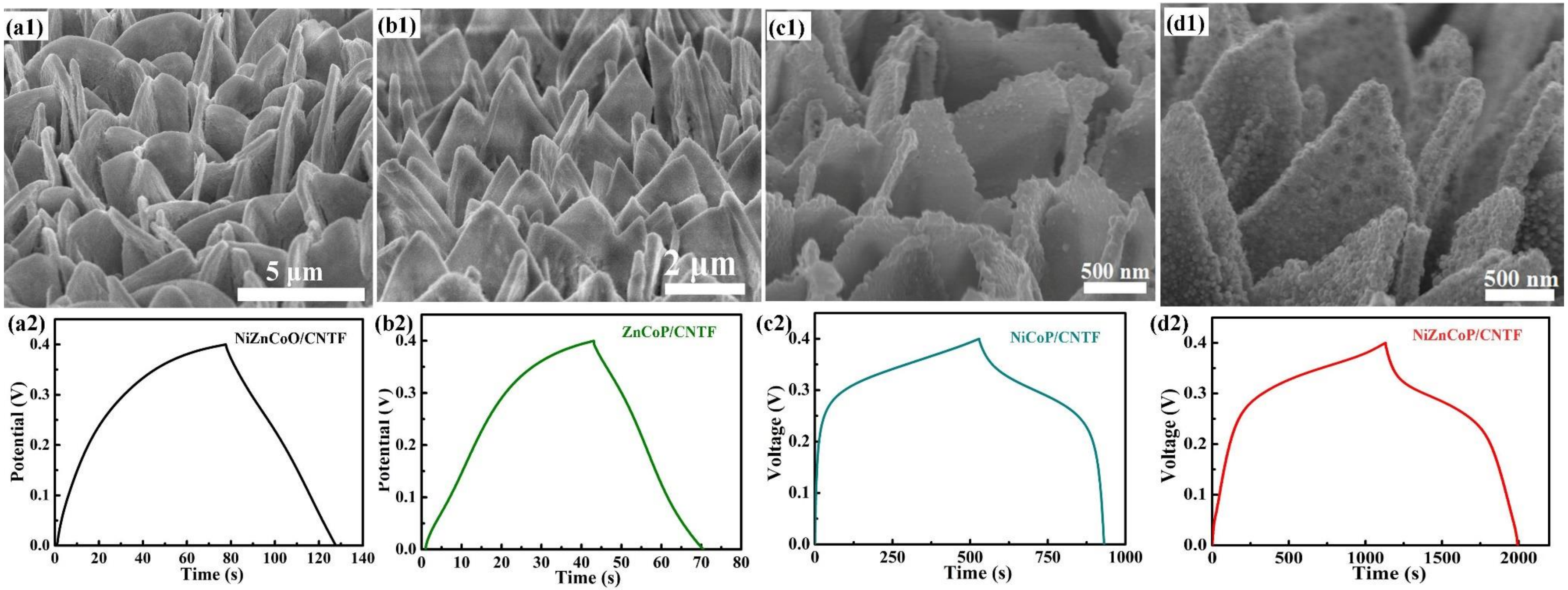

Figure S6. SEM images and GCD curves of NiCoP/CNTF and NiZnCoP/CNTF obtained at a current density of 1 mA/cm^2 of different cathodes. (a) NiZnCoO/CNTF; (b) ZnCoP/CNTF; (c) NiCoP/CNTF; (d) NiZnCoP/CNTF.

Figure S6a1-d1compare the SEM images of different cathodes, it is observed that the surface of the nanosheet arrays appears massive nanocrystalline when the Zn, Ni, Co, P, N, C co-existence in this material.

(1) Compared with Figure S6a2 and d2, it can be clearly seen that the cathode shows enhanced specific capacity and obvious discharge plateaus by replacing O element with P element.

(2) Compared with Figure S6b2 and d2, it can be clearly seen that the cathode shows enhanced specific capacity and obvious discharge plateaus by introducing Ni element.

(3) Compared with Figure S6c2 and d2, it can be clearly seen that the cathode shows enhanced specific capacity by introducing Zn element.

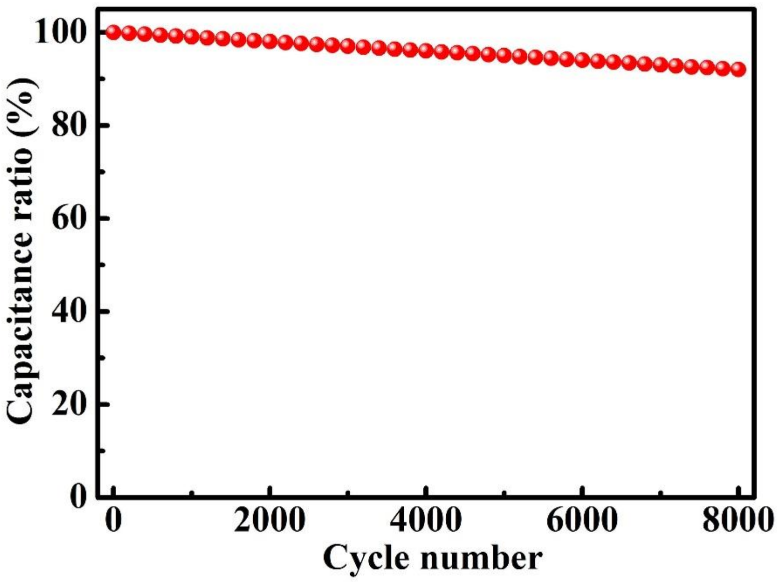

Figure S7. Cycling performance of the as-fabricated NiZnCoP/CNTF.

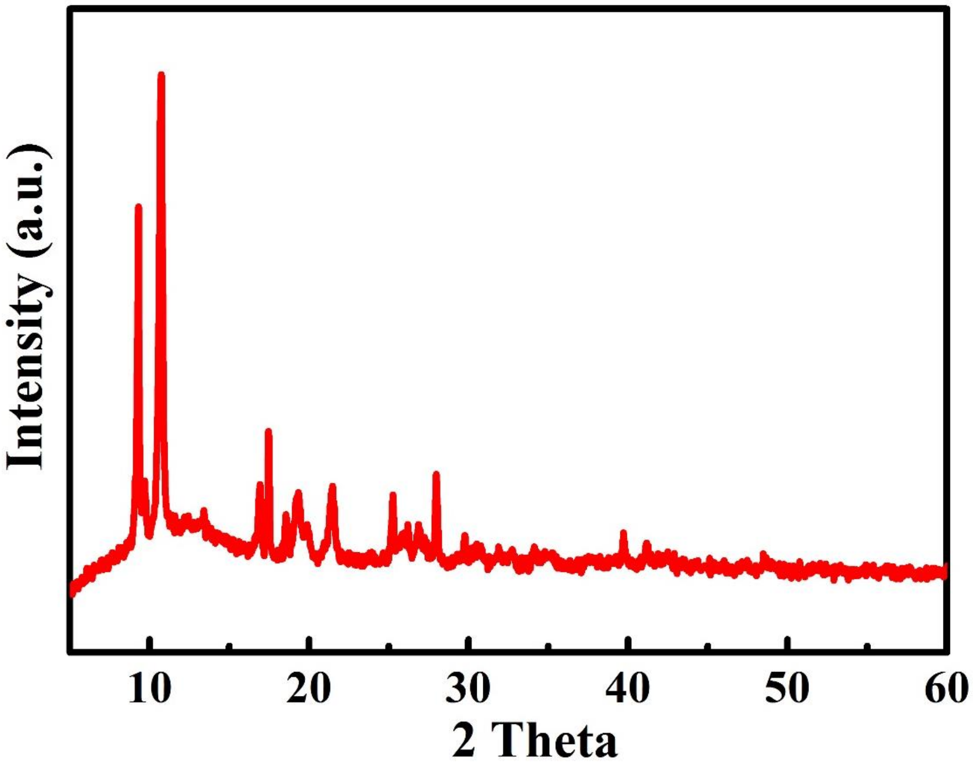

Figure S8. The XRD patterns of MIL-88-Fe.

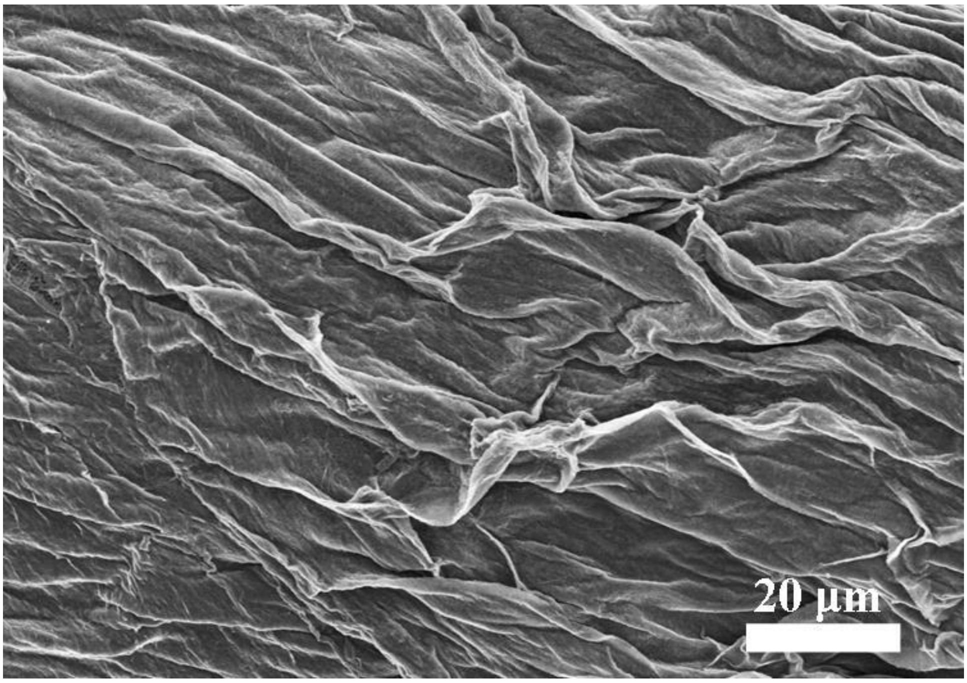

Figure S9. The SEM of the oxidized CNTF.

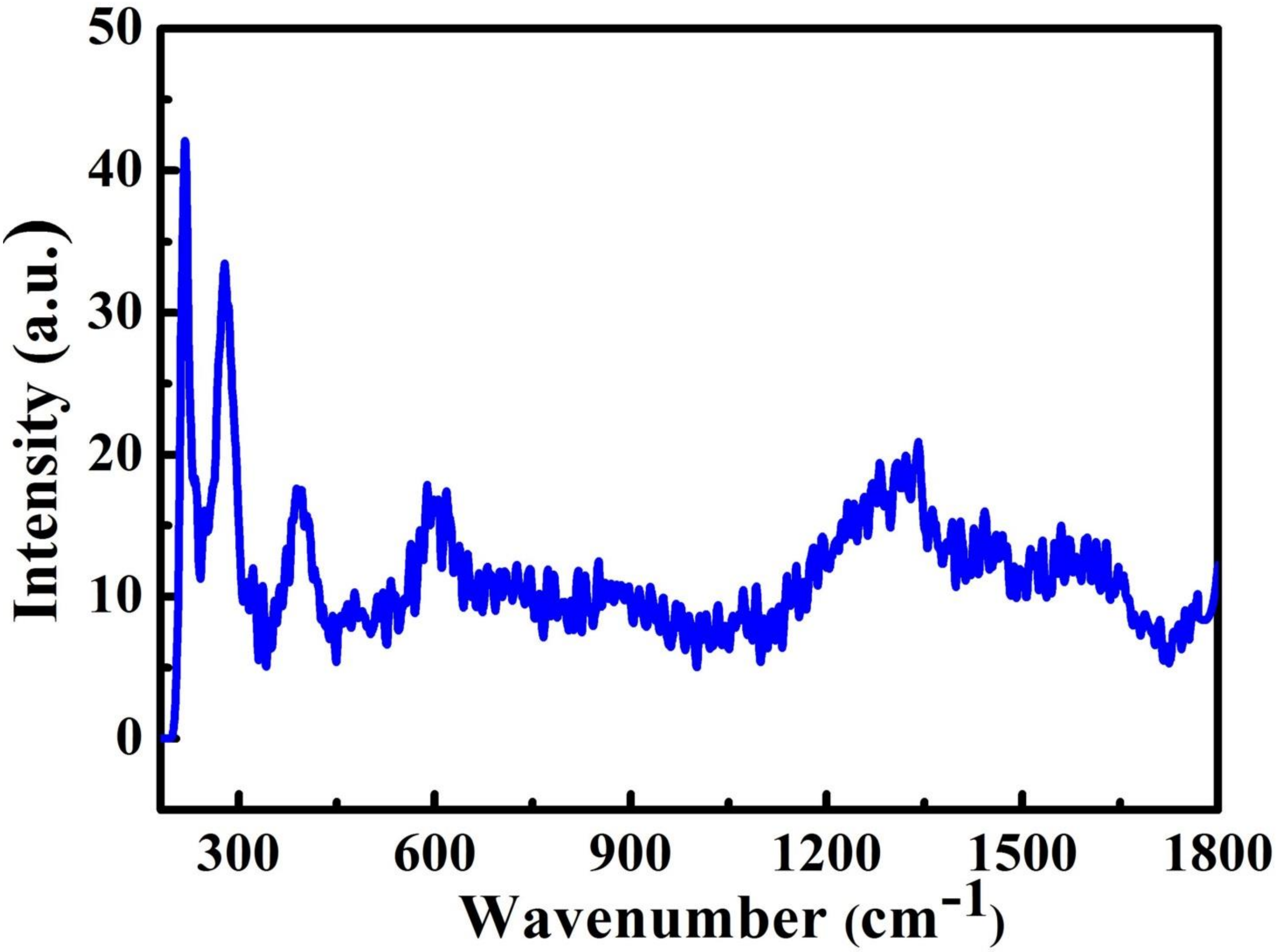

Figure S10. Raman spectroscopy of spindle-like α-Fe_2O_3.

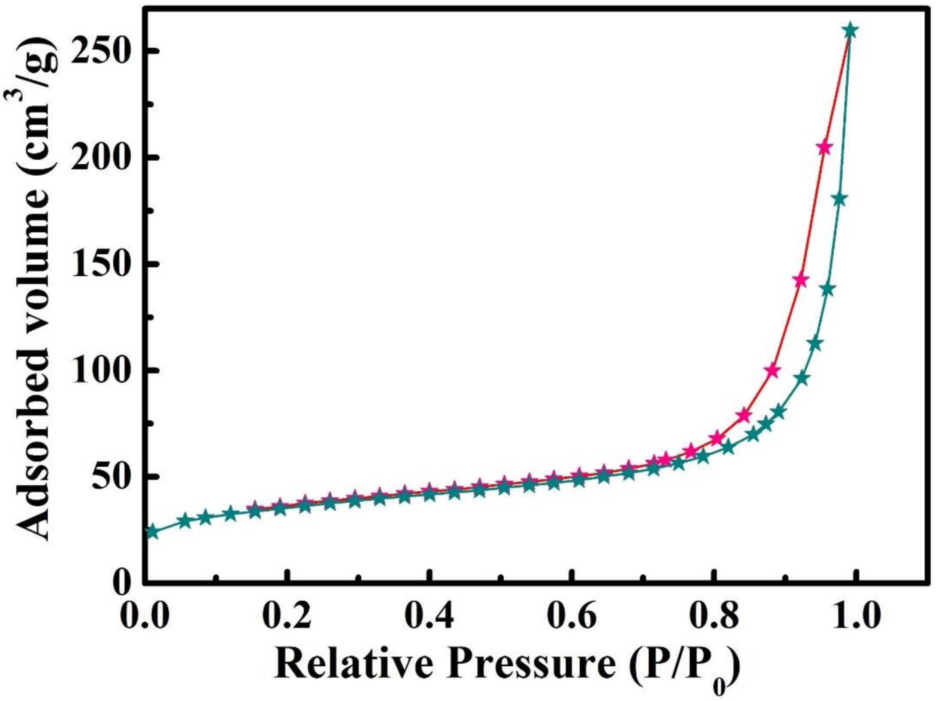

Figure S11. N_2 absorption-desorption isotherm of the α-Fe_2O_3 electrode materials.

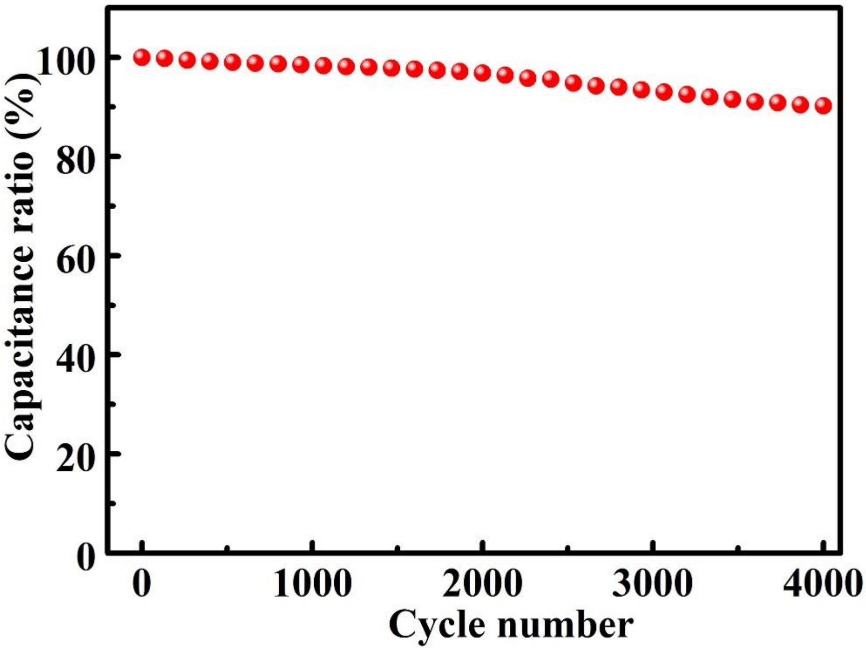

Figure S12. Cycling performance of the as-fabricated S-α-Fe_2O_3/OCNTF.

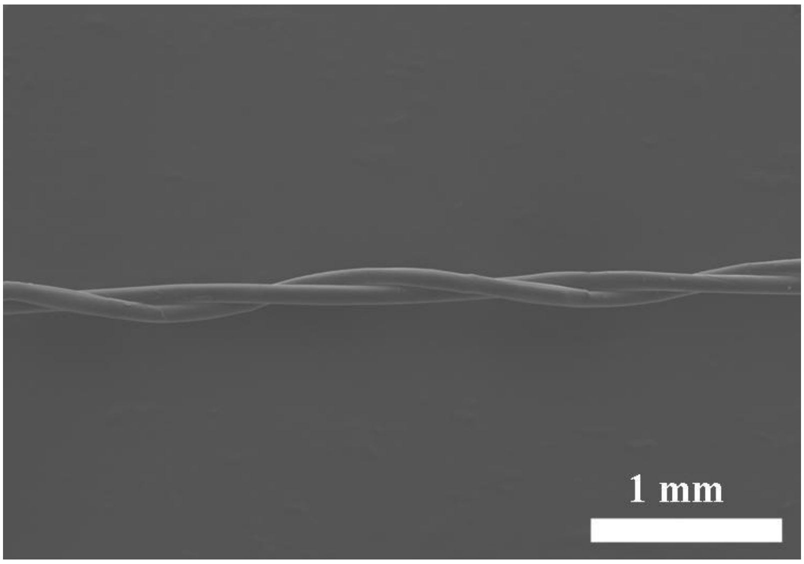

Figure S13. Low-magnification SEM image of our FARB device.

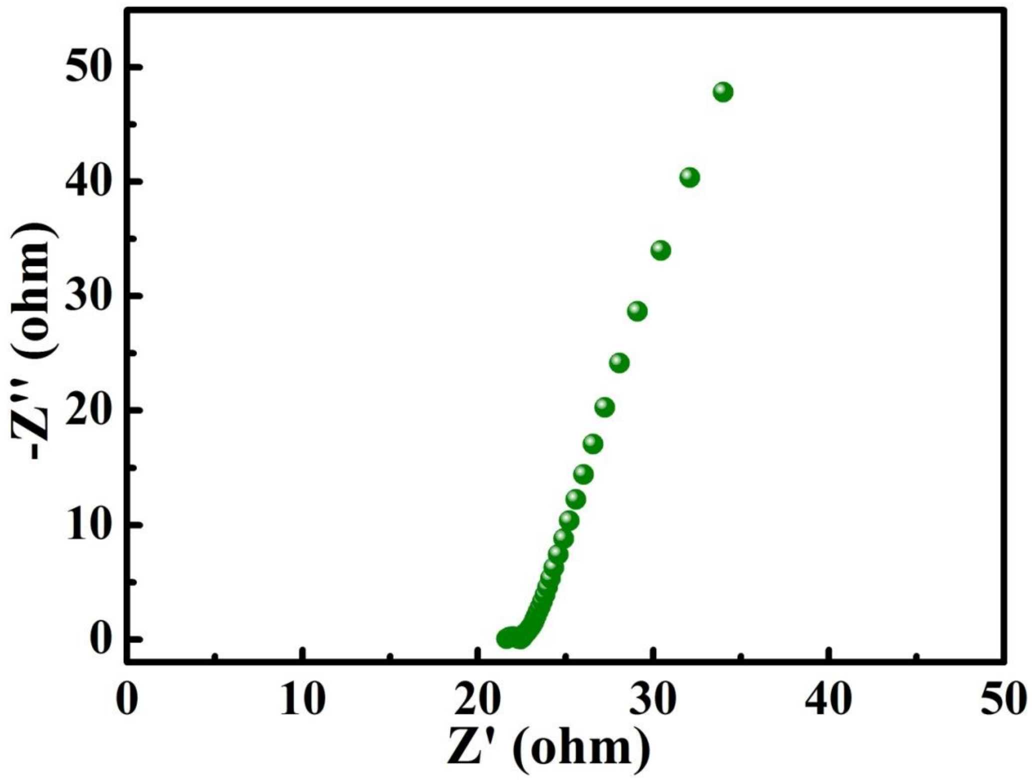

Figure S14. Nyquist plot of the as-prepared FARB at frequencies ranging from 10^{-2} to 10^{5} Hz at voltage amplitude of 5 mV.

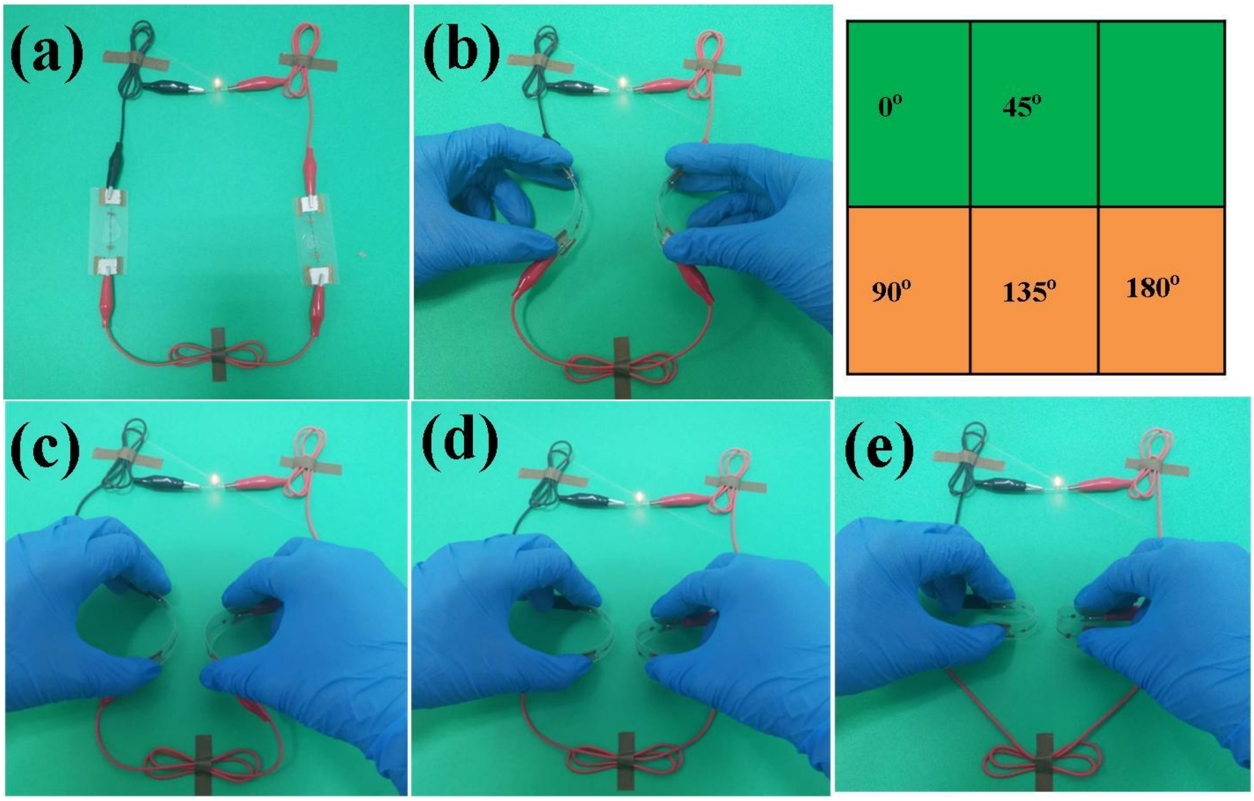

Figure S15. Representative digital image showing an LED illuminated by two series-connected FARBs in different bending angles.

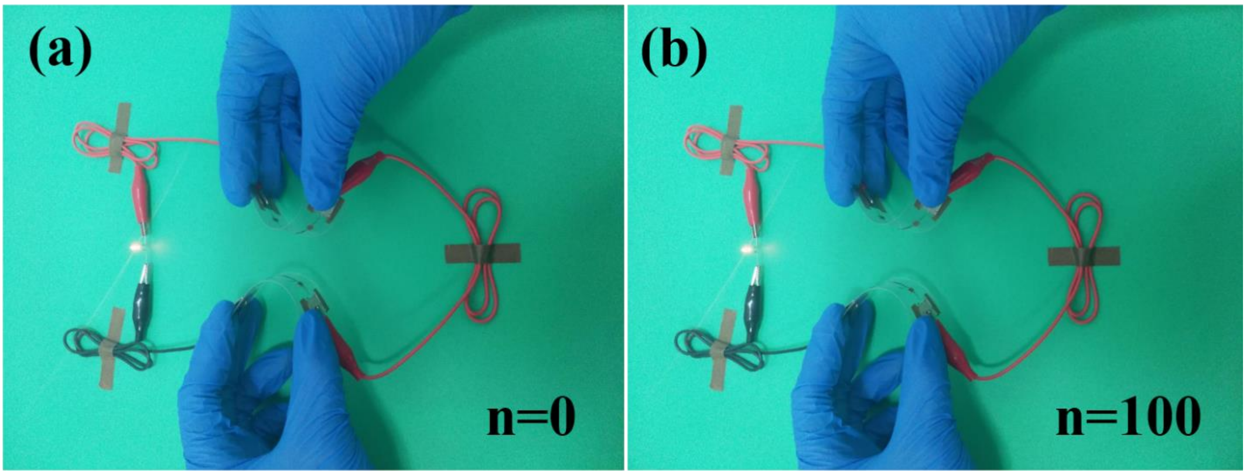

Figure S16. Representative digital image showing an LED illuminated by two series-connected FARBs with different bending cycles.

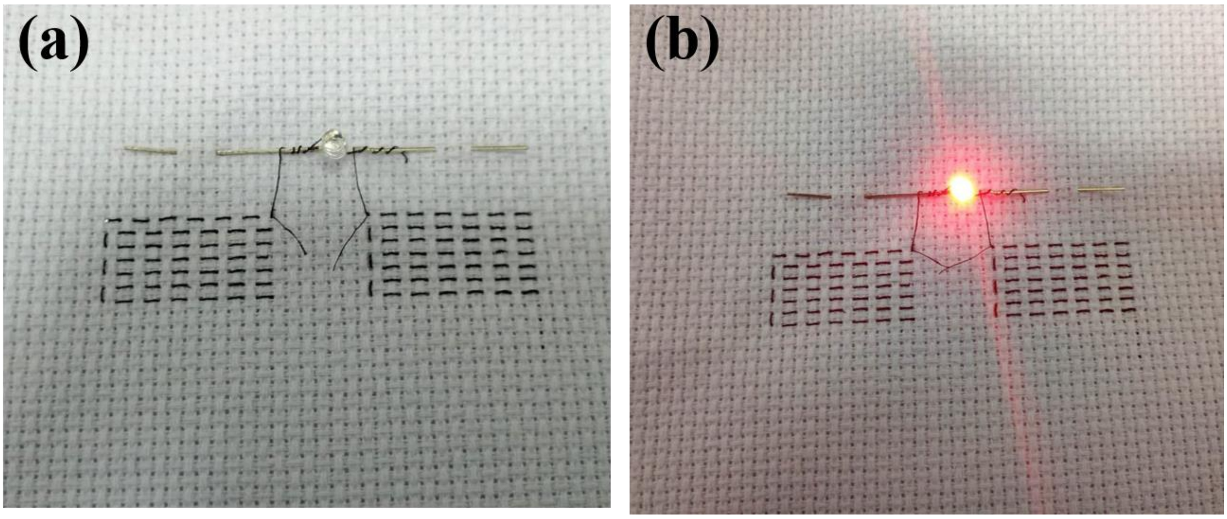

Figure S17. (a) The textiles of the FARBs device before illuming a LED. (b) The textiles of the FARBs device illume a LED.
